# Impact of dairy consumption on essential hypertension: a clinical study

**DOI:** 10.1186/1475-2891-13-83

**Published:** 2014-08-14

**Authors:** Jean-Philippe Drouin-Chartier, Iris Gigleux, André J Tremblay, Luc Poirier, Benoît Lamarche, Patrick Couture

**Affiliations:** Institute of Nutrition and Functional Foods (INAF), Pavillon des Services, Laval University, 2440, Hochelaga Blvd, Quebec City, G1V 0A6 Canada; CHUQ Research Center, Laval University, Quebec City, Canada

**Keywords:** Dairy products, Nutrition, Clinical trial, Essential hypertension, Ambulatory monitoring

## Abstract

**Background:**

Several studies have presented evidence suggesting that dairy consumption has beneficial effects on blood pressure (BP) in healthy subjects; however, only a few studies have examined this possibility in patients with established essential hypertension using ambulatory blood pressure monitoring. The objective of this study was to investigate how consuming dairy products impacts mean daytime systolic and diastolic BP in men and women with mild to moderate essential hypertension.

**Methods:**

Eighty-nine men and women with systolic BP ≥ 135 mm Hg and ≤ 160 mm Hg and diastolic BP ≤ 110 mm Hg were enrolled in this single-blind, randomized, cross-over, controlled study. Participants had to incorporate three daily servings of dairy products or control products equivalent in macronutrients and sodium during four-week treatment phases. Twenty-four hour ambulatory BP and endothelial function were assessed at screening and at the end of each dietary phase.

**Results:**

The consumption of three daily servings of dairy products led to a significant reduction in mean daytime ambulatory systolic BP (-2 mm Hg; P = 0.05) in men compared with readings after the control phase. In women, dairy consumption had no effect on ambulatory systolic BP. Moreover, endothelial function was significantly improved by dairy consumption in the whole cohort.

**Conclusion:**

These data indicate that the consumption of three daily servings of dairy products have beneficial effects on daytime systolic ambulatory BP compared to a heart-healthy, dairy-free, diet in men with mild to moderate essential hypertension.

**Trial registration:**

This trial is registered at clinicaltrials.gov as NCT01776216.

## Background

Arterial hypertension, which is defined as mean ambulatory daytime systolic blood pressure (BP) ≥ 135 mm Hg and diastolic BP ≥ 85 mm Hg, is one of the most important risk factors for cardiovascular and renal disease [1]. In 2000, approximately 26% of adults worldwide had hypertension, and its prevalence is expected to increase to 29% in 2025. Suboptimal control of BP is responsible for 13.5% of premature deaths and 6.0% of disability-adjusted life years [2, 3]. One of the first complications of hypertension is impaired endothelial function. Endothelial dysfunction is an early marker of vascular damage and increases the risk of coronary artery disease [4, 5]. These cardiovascular risk factors are closely related, and treating hypertension improves endothelial function [5]. As an important public health burden, promoting a healthy lifestyle and diet is essential.

Diet is a key factor for preventing and treating elevated BP [6, 7]. Healthy eating patterns, such as the Dietary Approaches to Stop Hypertension (DASH), vegetarian diets or the Mediterranean diet, have been shown to have beneficial effects in controlling BP [8–10]. High intakes of potassium, calcium and magnesium or low intakes of sodium and alcohol may also contribute at different levels to lower BP [11, 12].

Dairy products are part of the healthy eating patterns cited previously and contain large amounts of potassium, calcium and magnesium. Daily consumption of this food group appears to be beneficial for BP control. Recent meta-analyses [13, 14], including nearly 45, 000 and 57, 000 subjects, and systematic reviews of the literature on dairy products, BP and hypertension [15, 16] showed that consuming low-fat dairy products and milk consumption is inversely associated with the risk of hypertension. The combination of the micronutrient composition (calcium, magnesium and potassium) and the bioactive peptides (lactotripeptides) of dairy products could act synergistically, which would thus explain this protective effect [15, 16]. Because these aforementioned studies evaluated the association between dairy products and the risk of hypertension, the causal link between dairy consumption and BP reduction remains unclear. In fact, randomized clinical trials that have attempted to assess this relationship either were not designed to identify nutrients that lower BP or used small sample sizes [8, 17–19]. For example, in the original DASH study, the diet rich in fruits, vegetables and low-fat dairy products led to a greater reduction in BP than the control diet without dairy products [8]. However, in addition to including dairy products, there were also other important differences in nutritional composition that could have played a significant role in BP control. Therefore, the impact of dairy products *per se* was not individually characterized in the DASH study.

Finally, very few studies have assessed BP using ambulatory BP monitoring (ABPM). APBM is a proven and reliable method to assess BP because it reflects the actual BP more accurately than casual or in-office BP measurements and allows white coat syndrome, hidden hypertension or nocturnal hypertension to be detected that otherwise could not be detected with standard measurements [20, 21].

The objective of this study was to investigate how the consumption of three daily servings of dairy products impacts the mean daytime systolic and diastolic BP in men and women with mild to moderate essential hypertension. We hypothesized that consuming three servings of dairy products per day would significantly reduce the mean daytime systolic and diastolic BP as measured by 24-h ABPM. We have also examined how the consumption of three daily servings of dairy products impacts endothelial function. We hypothesized that dairy consumption would significantly improve the endothelial function as measured by digital pulse amplitude tonometry.

## Results and discussion

**Figure 1:**
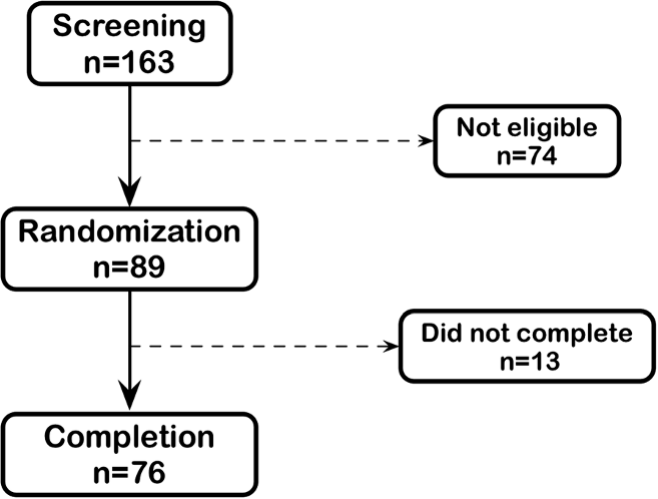
**Flow chart of participants.**

Of the 163 persons screened, 89 met the eligibility criteria and were randomized. During the study, 9 subjects dropped out of the protocol by their own and 4 were excluded for medical reasons (3 participants had sustained SBP > 160 mm Hg and 1 participant had a major surgery and general anesthesia). A total 76 participants (33 females and 43 males) completed the entire protocol (Figure 1). Demographic and anthropometric characteristics at the baseline (week 0) are presented in Table 1. The mean age and mean BMI of the participants were 53.3 ± 12.2 y and 28.2 ± 3.7 kg/m^2^, respectively. No significant differences in weight were noted at the end of each dietary intervention (Δ = 0.2 kg; P = 0.26). However, between the first and the last week of the CONTROL diet, a small but significant weight loss occurred (-0.3 ± 1.1 kg; P = 0.03). No such difference was measured between the first and the last week of the DAIRY diet (0.0 ± 1.0 kg; P = 0.70).

**Table 1:** **Baseline (week 0) characteristics of the participants who completed the study**

	n = 76
**Women (n)**	33
**Age (y)**	53.3 ± 12.2
**Weight (kg)**	80.6 ± 13.3
**Height (m)**	1.69 ± 0.11
**BMI (kg/m** ^**2**^ **)**	28.2 ± 3.7
**Waist circumference (cm)**	95.8 ± 11.1
**Office systolic BP (mm Hg)**	126 ± 11
**Office diastolic BP (mm Hg)**	81 ± 8
ABPM (Screening)	
**Daytime systolic BP**	144 ± 8
**Daytime diastolic BP**	87 ± 8
**24-h systolic BP**	140 ± 8
**24-h diastolic BP**	84 ± 7

Mean ± standard deviation.

Various methods were used to assess the compliance of participants to dietary intervention. First, an auto-reported checklist the participants completed daily allowed us to calculate the percentage of consumed food for each dietary treatment. The average compliance rates during the DAIRY and CONTROL treatment were 98.9 ± 1.8% and 98.7 ± 2.2%, respectively. Furthermore, the mean dairy intakes assessed using the FFQ were estimated at 3.4 (CI 95%: 2.9-3.9) servings/day during the DAIRY treatment and at 0.1 (0.0-0.1) servings/day during the CONTROL treatment. Finally, the 25(OH)D serum concentrations were significantly higher after the DAIRY phase than after the CONTROL phase (60.5 ± 18.8 nmol/ml vs. 53.5 ± 18.5 nmol/ml; P < 0.0001).

The consumption of three daily servings of dairy products led to no significant differences in daytime ambulatory systolic (Δ = -1 mm Hg; P_1_ = 0.38) and diastolic BP (Δ = 0 mm Hg; P_1_ = 0.59) compared with the values after the CONTROL phase (Table 2). However, a significant difference in endothelial function assessed by the reactive hyperemia index (RHI) was observed between the two interventions when adjusted for energy intake (DAIRY: 2.58 ± 0.52 vs. CONTROL: 2.50 ± 0.54; P_2_ = 0.04).

**Table 2:** **Ambulatory blood pressures (mm Hg) and RHI at the end of each dietary phase**

	Dairy	Control	Δ	P _1_	P _2_
**Daytime systolic BP**	142 ± 9	143 ± 9	-1 (-2 to 1)	0.38	0.45
**Daytime diastolic BP**	86 ± 9	86 ± 8	0 (-1 to 1)	0.59	0.42
**24-h systolic BP**	139 ± 9	139 ± 9	0 (-1 to 1)	0.68	0.81
**24-h diastolic BP**	83 ± 8	83 ± 8	0 (-1 to 1)	0.59	0.42
**RHI**	2.58 ± 0.52	2.50 ± 0.54	0.09 (-0.05 to 0.22)	0.21	0.04

Dairy and Control BP: Mean ± standard deviation.

Δ: Difference in mm Hg between Dairy and Control values (95% CI).

RHI: reactive hyperemia index.

P_1_: Unadjusted paired t-test.

P_2_: Adjusted for energy intake.

To address the potential role of various dietary factors on BP control, dietary composition was assessed using a standardized FFQ during the last week of each intervention phase (Table 3). During the DAIRY diet, participants consumed significantly less fruits and vegetables (-3.0; P < 0.0001) and meat and substitutes (-0.3; P = 0.001). Those differences were mainly attributable to the provided foods during the CONTROL diet, i.e. 3.3 servings of fruits and vegetables and 0.6 serving of meat and substitutes. Also, during the DAIRY diet, participants consumed significantly more sodium (238 mg/day; P = 0.008) and more calories (254 kcal/day; P = 0.0005), even if the dairy products and the control foods provided were matched for these nutrients. As expected, calcium, magnesium, potassium and vitamin D intakes were significantly higher during the DAIRY phase than during the CONTROL phase.

**Table 3:** **Dietary composition during each phase of treatment**

	Dairy	Control	Δ	P
**Dairy products (servings)**	3.4 (2.9-3.9)	0.1 (0.0-0.1)	3.3	<0.0001
**Fruits and vegetables (servings)**	7.0 (6.2-7.8)	10.0 (9.1-11.0)	-3.0	<0.0001
**Meat and substitutes (servings)**	2.5 (2.2-2.7)	2.8 (2.6-3.1)	-0.3	0.001
**Cereals (servings)**	4.4 (4.0-4.9)	4.1 (3.7-4.5)	0.3	0.11
**Energy (kcal)**	2239 (2089-2401)	1985 (1827-2157)	254	0.0005
**Alcohol (%)**	0.6 (0.4-1.1)	0.8 (0.5-1.2)	-0.2	0.35
**Lipids (%)**	32.1 (31.1-33.3)	32.5 (31.5-33.6)	-0.4	0.54
**SFA (%)**	10.7 (10.3-11.1)	8.1 (7.8-8.4)	2.6	<0.0001
**MUFA (%)**	12.9 (12.4-13.5)	14.2 (13.6-14.8)	-1.3	<0.0001
**PUFA (%)**	5.8 (5.5-6.2)	7.3 (7.0-7.6)	-1.4	<0.0001
**TFA (%)**	1.1 (1.0-1.2)	1.1 (1.1-1.2)	0.0	0.61
**Dietary cholesterol (mg)**	254 (232–278)	204 (182-228)	50	<0.0001
**Protein (%)**	17.6 (17.0-18.1)	14.4 (13.8-15.0)	3.2	<0.0001
**Carbohydrates (%)**	50.4 (49.2-51.6)	54.2 (52.6-55.7)	-3.8	<0.0001
**Fiber (g)**	24.9 (22.8-27.2)	26.1 (23.9-28.5)	-1.2	0.21
**Sodium (mg)**	2729 (2533-2941)	2491 (2307-2691)	238	0.008
**Calcium (mg)**	1492 (1376-1617)	533 (481-591)	959	<0.0001
**Magnesium (mg)**	445 (415-477)	410 (379-444)	35	<0.0001
**Potassium (mg)**	4219 (3956-4500)	3830 (3548-4134)	389	0.01
**Vitamin D (μg)**	9.7 (8.9-10.6)	4.9 (4.2-5.9)	4.7	0.006

73 participants completed the FFQ after both treatment.

Geometric mean (95% CI).

Δ: Difference between Dairy and Control values.

**Figure 2:**
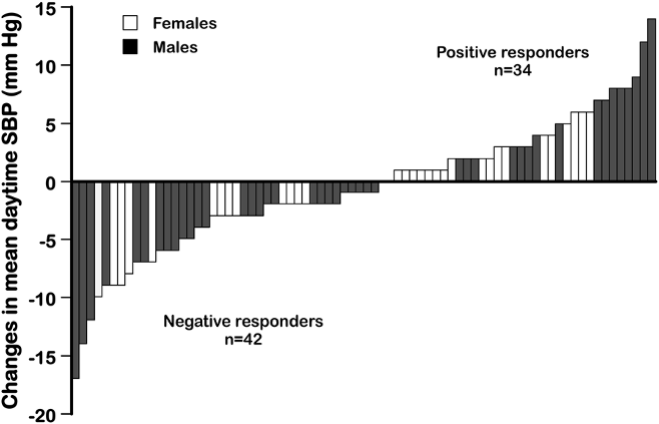
**Individual variation In mean daytime systolic BP between the DAIRY diet and the CONTROL diet.** Each bar represents the change (mm Hg) in blood pressure for one study subject; these data are arranged in rank to show the range of variation.

Figure 2 presents the variation in daytime systolic BP between both treatments for each participants. BP variation was calculated as the difference in the mean daytime systolic BP between the DAIRY and CONTROL diets. The mean daytime systolic BP was lowered by dairy consumption for 40 subjects and remained stable or increased for 36 participants. The relative proportion of men tended to be higher among negative responders than among positive responders (0.65 vs 0.44; P = 0.06). Further analyses also suggested a difference in SBP change between men and women after the dairy phase (Interaction test: treatment*sex, P = 0.07). Consequently, the impact of dairy consumption on BP and endothelial function was assessed in men and women separately. The consumption of three daily servings of dairy products led to a significant reduction in mean daytime ambulatory systolic BP (-2 mm Hg; P = 0.05) in men compared with readings after the CONTROL phase (Table 4). In women, dairy consumption had no significant impact on systolic BP and endothelial function (Table 5). The diastolic BP was higher after the DAIRY phase than after the CONTROL phase (1 mm Hg; P = 0.05).

**Table 4:** **Ambulatory BP (mm Hg) and RHI at the end of each dietary phase for men (n = 43)**

	Dairy	Control	Δ	P
**Daytime systolic BP**	142 ± 10	144 ± 10	-2 (-3 to 0)	0.05
**Daytime diastolic BP**	87 ± 9	88 ± 8	-1 (-2 to 1)	0.37
**24 h systolic BP**	138 ± 9	139 ± 9	-1 (-3 to 0)	0.12
**24 h diastolic BP**	84 ± 8	84 ± 8	0 (-1 to1)	0.61
**RHI**	2.41 ± 0.43	2.30 ± 0.42	0.11 (-0.06 to 0.28)	0.63

Mean ± standard deviation.

Δ: Difference in mm Hg between Dairy and Control values (95% CI).

**Table 5:** **Ambulatory BP (mm Hg) and RHI at the end of each dietary phase for women (n = 33)**

	Dairy	Control	Δ	P
**Daytime systolic BP**	142 ± 9	142 ± 8	0 (-1 to 3)	0.45
**Daytime diastolic BP**	85 ± 9	84 ± 8	1 (0 to 2)	0.05
**24 h systolic BP**	139 ± 8	138 ± 7	1 (-1 to 3)	0.30
**24 h diastolic BP**	82 ± 8	81 ± 8	1 (0 to 2)	0.13
**RHI**	2.83 ± 0.52	2.78 ± 0.55	0.05 (-0.18 to 0.29)	0.63

Mean ± standard deviation.

Δ: Difference in mm Hg between Dairy and Control values (95% CI).

This study evaluated the impact of consuming three daily servings of dairy products on BP in men and women with mild to moderate essential hypertension. This study is one of the first to assess the impact of dairy consumption *per se* on BP using ABPM with a considerable sample size (n = 76). Consumption of dairy products corresponding to the current Canadian recommendations, in the context of a heart healthy diet, led to a significant improvement in the endothelial function in the whole cohort. Also, a reduction in mean daytime systolic BP was observed in men only. In women, dairy consumption had no significant impact on ambulatory systolic BP.

In the DASH study, the combination diet (or DASH diet), which included adequate amounts of low-fat dairy products in addition to a higher amount of fruits, vegetables and reduced amounts of saturated and total fat, was associated with significant reductions in mean 24-h systolic and diastolic BP, as measured by ABPM [8]. The effects of this diet on the 24-h systolic and diastolic BP were more important than the effects of the control diet and the fruits and vegetables diet, both of which excluded dairy products, and were greater among hypertensive subjects than among normotensive subjects. In the present study, the absence of a significant impact of the intake of three daily dairy servings on BP control in the whole cohort of hypertensive subjects could be related, at least in part, to important differences in the diet compositions between the two intervention treatments.

The first difference regarding diet composition is related to the daily consumption of sodium, which was significantly higher during the DAIRY phase than during the CONTROL phase (+238 mg; P = 0.008), despite the fact that the DAIRY and CONTROL products had the same sodium content. A high consumption of sodium has been shown to have a deleterious impact on BP control [19, 22]. Based on the DASH sodium study [19], we assessed that this difference in sodium intake had an impact of 0.6 mm Hg on systolic BP and of 0.3 mm Hg on diastolic BP. Therefore, it is likely that the higher intake of sodium might have attenuated the beneficial effect of dairy consumption on BP.

Another important consideration in the interpretation of these results is related to the daily consumption of fruits and vegetables. In fact, consumption of fruits and vegetables was lower during the DAIRY phase than during the CONTROL phase (-3.0 servings/day; P < 0.0001). A diet rich in fruits and vegetables has been shown to exert beneficial effects on BP control. In the DASH study, the diet rich in fruits and vegetables, which contained 8.5 servings of fruits, juice and vegetables and no dairy products was associated with significant reductions in both systolic and diastolic BP compared with the control diet that was poor in fruits, juice and vegetables (3.6 servings/day) [8]. Based on these data, we assessed that the difference in the fruits and vegetables intake observed in the current study might have an impact up to 1.7 mm Hg on SBP and 0.7 mm Hg on DBP. It is important to emphasize that the 3.0 serving difference in daily consumption of fruits and vegetables observed in the current study was mainly constituted of juices and that the effect on BP might be lower than expected based on the DASH study. Nonetheless, it is likely that the higher consumption of fruits and vegetables, caused by the provided control foods may have had favorable effects on BP during the CONTROL phase.

Previous studies have also shown that weight loss is associated with beneficial effects on BP control. A meta-analysis reported that a 1-kg reduction in body weight is associated with a reduction of 1.1 mm Hg in systolic BP and 0.9 mm Hg in diastolic BP [23]. The relationship between weight loss and BP reduction appears to be linear [24]. In the present study, the significant weight loss might have lowered both SBP and DBP of 0.3 mm Hg during the CONTROL treatment.

Taken together, the combined effects of the lower sodium intake (-0.5/-0.3 mm Hg), the consumption of more fruits and vegetables (-1.7/-0.7 mm Hg) and the significant weight loss (-0.3/-0.3 mm Hg) during the CONTROL phase may have decreased BP by 2.5/1.3 mm Hg and therefore attenuated the impact of dairy consumption by reducing the difference in BP between each treatment phase.

Our results, showing that dairy consumption improves BP control in men, support the concept that constitutional factors could play a role in the heterogeneity of BP response to dairy intake. Several studies have suggested that sex steroid hormones have direct vascular effects that may contribute to the gender differences in BP regulation [25, 26]. Interestingly, these results are in contrast with those from the DASH study in which the DASH diet compared with the Fruits and Vegetables diet significantly reduced BP in women, but not in men [8]. Our results also suggested that dairy consumption could have a detrimental effect on DBP in women. Further studies are clearly needed to assess the contribution of gender-related factors to the BP response to dairy intake.

The short duration of the intervention periods and the partly controlled diet are the two limitations of this study. In addition, as the study population included subjects with mild to moderate hypertension, our results cannot be extrapolated to individuals with severe hypertension that may have a better BP response to dairy products [8]. Furthermore, it is important to note that the use of ABPM versus the standard sphygmomanometer measurement taken in a doctor’s office has most likely enhanced the validity of the BP measurements in the present study [27]. Several lines of evidence indicate that ABPM data, particularly the mean daytime systolic BP, correlate more closely than conventional in-office measurements with target organ injury [28] and CVD risk [29].

## Conclusions

The results of the current study suggest that dairy consumption has beneficial effect on endothelial function of mild to moderate hypertensive subjects. Also, our results indicate that the daily consumption of three servings of dairy products have beneficial impacts on daytime systolic BP, compared to a heart-healthy diet that excludes dairy, in mild to moderate hypertensive men. In addition, our results suggest that gender could play a role in the heterogeneity of BP response to dairy intake. Finally, the lack of a response to dairy intake in the whole cohort may be related to the lower sodium, significant weight loss and increased consumption of fruit and vegetables in the control diet. Therefore, controlled studies in which all foods are provided to participants to minimize interindividual variations in dietary composition are required to assess the independent contribution of dairy intake on BP control in hypertensive subjects.

## Methods

### Population

Eighty-nine adults were recruited between August 2011 and December 2012 in Quebec City at the Institute of Nutrition and Functional Foods (INAF) of Laval University. To be part of the study, participants had to be aged between 18–70 years old, have mild to moderate essential hypertension (mean daytime systolic BP ≥ 135 mm Hg and ≤ 160 mm Hg and mean daytime diastolic BP ≤ 110 mm Hg, as identified with 24-h ABPM) and have maintained a stable body weight for at least six months prior to the study. Participants with body mass indices (BMI) > 35 kg/m^2^, that were smokers (>1 cigarette/day), with a previous history of cardiovascular disease, with type 2 diabetes, with monogenic dyslipidemia, that were taking anti-inflammatory drugs, that had endocrine or gastrointestinal disease, that had an allergy to dairy, that were clinically using vitamin D or calcium supplements, that were vegetarians or that had any other conditions that may interfere with optimal participation in the study were ineligible.

Subjects taking anti-hypertensive drugs were eligible; however, they had to stop taking their medication at least three weeks before screening and for the duration of the study, under the approval of the study physician. Subjects taking lipid-lowering drugs were eligible under the same condition; however, they had to stop taking their medication at least four weeks before screening.

Pre-menopausal women were eligible, irrespective of the use of contraceptive agents, if their menstrual cycle had been regular for the last three months (25–35 days/cycle). Post-menopausal women were eligible; however, their hormone supplementation status had to remain constant for the duration of the study. Women who started hormone replacement therapy within six months prior to the study were ineligible. Finally, all participants signed an informed consent document before being enrolled. The project was approved by the Laval University Ethical Review Committee.

### Study design

**Figure 3:**
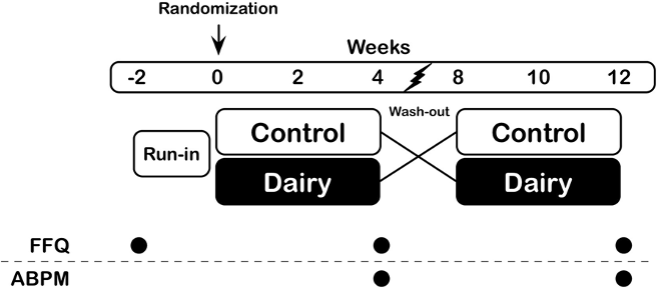
**Study design (FFQ: food frequency questionnaire; ABPM: ambulatory blood pressure monitoring).**

In this single-blind, randomized, cross-over, controlled study, participants who met the inclusion criteria first received nutritional advice to adapt their diet to a prudent dietary pattern (25-35% of calories from fat, <10% of calories from saturated fatty acids, <1% of calories from *trans* fatty acids, dietary cholesterol <200 mg/d and sodium <2300 mg/d). A trained registered dietitian gave recommendations that were based on a validated self-administered food frequency questionnaire (FFQ), which assessed the participants’ diets from the preceding four weeks [30] and from three-day food diaries. A two-week run-in period followed to allow participants to familiarize themselves with the recommendations (Figure 3).

After the run-in period, participants were randomly assigned to a DAIRY-CONTROL or a CONTROL-DAIRY diet sequence using computer-generated numbers. Each dietary phase lasted four weeks separated by a four-week wash-out period. During the wash-out period, participants were told to continue with the dietary recommendations they received during the run-in period. The run-in period refers the period between week -2 and week 0, and the baseline refers to week 0. Weeks 0 to 4 and 8 to 12 were the two treatment periods, and the time lapse between week 4 and week 8 was the wash-out period (Figure 3). During the DAIRY diet, participants had to incorporate the equivalent of 3.1 daily servings of dairy products into their normal day diet. During the CONTROL diet, they had to incorporate control products equivalent in energy, saturated fatty acids (SFA) and sodium content. Participants had to avoid consuming any dairy products during the CONTROL period. The food types, serving sizes and nutritional composition of each treatment are presented in Table 6. All dairy products or control foods were provided to participants on a weekly basis. Behavioral and psychological counselling was offered to subjects throughout the study to maximize the success of the nutritional changes and to ensure their compliance with treatment.

**Table 6:** **Nutritional composition of dairy and control foods**

	Dairy diet	Control diet
**Food**	Milk, 1% m.f., 375 ml	Fruit juice, 290 ml
	Cheddar cheese, 34% m.f., 30 g	Vegetable juice, 156 ml
	Stirred yogurt, 1.5% m.f., 175 g	Salted cashews, 20 g
		Cookie (homemade), 1
**Energy (kcal)**	432	438
**Proteins (g)**	26.0	7.7
**Carbohydrates (g)**	45.0	63.3
**Fibers (g)**	0.0	3.2
**Lipids (g)**	16.3	17.1
**SFA (g)**	8.7	7.6
**MUFA (g)**	4.11	6.6
**PUFA (g)**	0.42	1.91
**Sodium (mg)**	465	459
**Calcium (mg)**	913	84

### Concealment

Study participants and study coordinators were not blind because of the tangibility of the study food (see Table 6). However, investigators and laboratory staff were blinded until the final statistical analyses were conducted.

### Ambulatory BP monitoring

Twenty-four-hour ABPM was performed using a Spacelabs 90207 device (Spacelabs Inc., Redmond, WA) at the screening and at the end of each dietary phase. The daytime period was set from 6:00 AM to 10:00 PM. During the daytime, BP was measured every 20 minutes, and during the night, every 30 minutes. Participants had to wear the device for all of the time (24 hours) and had to complete a physical activity diary in parallel to assess their daily energy expenditure [31]. To be considered valid, the percentage of valid readings of each ABPM session had to be no less than 70%.

### Office BP measurement

On a weekly basis, study subjects had to visit the research center for BP measurement. Auscultatory readings of BP were performed using a properly calibrated, automatic BP monitor (BP Thru, Omron) and were supervised by a nurse or a research professional. Subjects were asked to avoid caffeine 30 minutes before the appointment and had to sit quietly for 10 minutes before beginning the measurements. A minimum of three sequential readings were taken with three minutes between readings.

### Dietary assessment

Before the run-in period and at weeks 4 and 12, participants had to complete a three-day food diary and a validated web auto-administered FFQ [30], which assessed their food intake from the preceding four weeks and therefore, over the intervention periods.

### Endothelial function assessment

Endothelial function was assessed via digital pulse amplitude tonometry using an Endo-PAT2000 device (Itamar Medical, Caesarea, Israel) at the end of each dietary phase. This device uses non-invasive technology to measure the reactive hyperemia following an ischemia in the forearm. Patients were placed in a horizontal position. Two finger-mounted probes were attached to the distal phalanx of their index finger, which measured the arterial pulsatile volume change. A BP cuff was placed on the non-dominant forearm, and the other forearm served as the control. After a five-minute baseline period, the cuff inflated at a 250 mm Hg pressure that created an ischemia in the study forearm. After five minutes, the cuff was deflated, and the pulse amplitude tonometry was recorded for the last five-minute period. The outcome measurement that assesses the endothelial function is the reactive hyperemia index (RHI), which is the ratio of the digital pulse volume during reactive hyperemia compared with the baseline digital pulse volume. This method has been validated, is user-independent and gives a similar result as the flow-mediated dilation method, which also assesses the endothelial function [32, 33].

### Compliance assessment

Compliance was evaluated using checklists that the participants completed daily. Information from the three-day food diaries, FFQ and 25-OH vitamin D serum concentration obtained or measured at the end of each dietary phase were used in addition to the checklists to assess their compliance with the dietary advice given before the run-in period and the treatment foods during the intervention periods.

### Anthropometry

Anthropometric measurements (body weight, waist circumference and hip circumference) were collected at the screening and at weeks 0, 2, 4, 8, 10 and 12. Body weight had to remain constant for the duration of the study.

### Statistical analyses

The primary study outcome is the difference in the mean daytime systolic BP between the DAIRY and the CONTROL diets. The change in the mean daytime diastolic BP is the secondary study outcome and the third outcome is the difference in the RHI between both dietary phases. Unadjusted paired t-test and PROC mixed procedures for repeated measurements with adjustment for significant covariable were performed to compare the difference between the mean daytime blood pressure and RHI in the whole cohort and for gender using JMP software (v10.0.0, Cary, NC) and SAS software (v9.3, Cary, NC). Assessment of any interaction and adjustments for significant covariables through multivariate modeling were performed using the same procedure. Differences with P ≤ 0.05 were considered statistically significant. Non-normally distributed data were log transformed prior to the analyses.

### Sample size estimate

The study was designed to accurately test our main hypothesis. We performed our calculations based on data presented in the original DASH study [8], in which 354 of the 459 subjects had their BP assessed with 24-h ABPM and in which 50-60% of the change in BP was attributable to dairy consumption *per se* [34]. We determined that in our study, having 80 subjects complete the two phases of the intervention would allow us to detect a clinically meaningful 5 mm Hg change in mean daytime systolic BP with a power of 90% and a clinically meaningful 3 mm Hg change in mean daytime diastolic BP with a power of 90%. To account for the anticipated 15% drop out, we attempted to recruit 92 subjects, with equal numbers of males and females.

## Authors’ information

Benoît Lamarche is the recipient of a Chair in Nutrition and Cardiovascular Health at Laval University. PC is professor of medicine at Laval University.

## Abbreviations

ABPM: Arterial blood pressure monitoring
BMI: Body mass index
BP: Blood pressure
FFQ: Food frequency questionnaire.

## References

[1] Go AS, Mozaffarian D, Roger VL, Benjamin EJ, Berry JD, Borden WB, Bravata DM, Dai S, Ford ES, Fox CS, Franco S, Fullerton HJ, Gillespie C, Hailpern SM, Heit JA, Howard VJ, Huffman MD, Kissela BM, Kittner SJ, Lackland DT, Lichtman JH, Lisabeth LD, Magid D, Marcus GM, Marelli A, Matchar DB, McGuire DK, Mohler ER, Moy CS, Mussolino ME (2013). Heart disease and stroke statistics–2013 update: a report from the American Heart Association. Circulation.

[2] Lawes CM, Vander Hoorn S, Rodgers A (2008). Global burden of blood-pressure-related disease, 2001. Lancet.

[3] Kearney PM, Whelton M, Reynolds K, Whelton PK, He J (2004). Worldwide prevalence of hypertension: a systematic review. J Hypertens.

[4] Munzel T, Sinning C, Post F, Warnholtz A, Schulz E (2008). Pathophysiology, diagnosis and prognostic implications of endothelial dysfunction. Ann Med.

[5] Bolad I, Delafontaine P (2005). Endothelial dysfunction: its role in hypertensive coronary disease. Curr Opin Cardiol.

[6] Sacks FM, Campos H (2010). Dietary therapy in hypertension. N Engl J Med.

[7] Zhao D, Qi Y, Zheng Z, Wang Y, Zhang XY, Li HJ, Liu HH, Zhang XT, Du J, Liu J (2011). Dietary factors associated with hypertension. Nat Rev Cardiol.

[8] Appel LJ, Moore TJ, Obarzanek E, Vollmer WM, Svetkey LP, Sacks FM, Bray GA, Vogt TM, Cutler JA, Windhauser MM, Lin PH, Karanja N (1997). A clinical trial of the effects of dietary patterns on blood pressure: DASH Collaborative Research Group. N Engl J Med.

[9] Estruch R, Martínez-González MA, Corella D, Salas-Salvadó J, Ruiz-Gutiérrez V, Covas MaI, Fiol M, Gómez-Gracia E, López-Sabater MC, Vinyoles E, Arós F, Conde M, Lahoz C, Lapetra J, Sáez G, Ros E (2006). Effects of a Mediterranean-Style Diet on Cardiovascular Risk FactorsA Randomized Trial. Ann Intern Med.

[10] Pettersen BJ, Anousheh R, Fan J, Jaceldo-Siegl K, Fraser GE (2012). Vegetarian diets and blood pressure among white subjects: results from the Adventist Health Study-2 (AHS-2). Public Health Nutr.

[11] Savica V, Bellinghieri G, Kopple JD (2010). The effect of nutrition on blood pressure. Annu Rev Nutr.

[12] Appel LJ, Brands MW, Daniels SR, Karanja N, Elmer PJ, Sacks FM (2006). Dietary approaches to prevent and treat hypertension: a scientific statement from the American Heart Association. Hypertension.

[13] Soedamah-Muthu SS, Verberne LD, Ding EL, Engberink MF, Geleijnse JM (2012). Dairy consumption and incidence of hypertension: a dose–response meta-analysis of prospective cohort studies. Hypertension.

[14] Ralston RA, Lee JH, Truby H, Palermo CE, Walker KZ (2012). A systematic review and meta-analysis of elevated blood pressure and consumption of dairy foods. J Hum Hypertens.

[15] McGrane MM, Essery E, Obbagy J, Lyon J, Macneil P, Spahn J, Van Horn L (2011). Dairy consumption, blood pressure, and risk of hypertension: an evidence-based review of recent literature. Curr Cardiovasc Risk Rep.

[16] Alvarez-Leon EE, Roman-Vinas B, Serra-Majem L (2006). Dairy products and health: a review of the epidemiological evidence. Br J Nutr.

[17] Kynast-Gales SA, Massey LK (1992). Effects of dietary calcium from dairy products on ambulatory blood pressure in hypertensive men. J Am Diet Assoc.

[18] Hilpert KF, West SG, Bagshaw DM, Fishell V, Barnhart L, Lefevre M, Most MM, Zemel MB, Chow M, Hinderliter AL, Kris-Etherton PM (2009). Effects of dairy products on intracellular calcium and blood pressure in adults with essential hypertension. J Am Coll Nutr.

[19] Sacks FM, Svetkey LP, Vollmer WM, Appel LJ, Bray GA, Harsha D, Obarzanek E, Conlin PR, Miller ER, Simons-Morton DG, Karanja N, Lin PH (2001). Effects on blood pressure of reduced dietary sodium and the Dietary Approaches to Stop Hypertension (DASH) diet: DASH-Sodium Collaborative Research Group. N Engl J Med.

[20] White WB (2003). Ambulatory blood-pressure monitoring in clinical practice. N Engl J Med.

[21] Marchiando RJ, Elston MP (2003). Automated ambulatory blood pressure monitoring: clinical utility in the family practice setting. Am Fam Physician.

[22] Aburto NJ, Ziolkovska A, Hooper L, Elliott P, Cappuccio FP, Meerpohl JJ (2013). Effect of lower sodium intake on health: systematic review and meta-analyses. BMJ.

[23] Neter JE, Stam BE, Kok FJ, Grobbee DE, Geleijnse JM (2003). Influence of weight reduction on blood pressure: a meta-analysis of randomized controlled trials. Hypertension.

[24] Stevens VJ, Obarzanek E, Cook NR, Lee IM, Appel LJ, Smith West D, Milas NC, Mattfeldt-Beman M, Belden L, Bragg C, Millstone M, Raczynski J, Brewer A, Singh B, Cohen J (2001). Long-term weight loss and changes in blood pressure: results of the Trials of Hypertension Prevention, phase II. Ann Intern Med.

[25] Barbagallo M, Dominguez LJ, Licata G, Ruggero R, Lewanczuk RZ, Pang PK, Resnick LM (2001). Effect of testosterone on intracellular Ca++ in vascular smooth muscle cells. Am J Hypertens.

[26] Barbagallo M, Dominguez LJ, Licata G, Shan J, Bing L, Karpinski E, Pang PK, Resnick LM (2001). Vascular effects of progesterone: role of cellular calcium regulation. Hypertension.

[27] Ohkubo T, Kikuya M, Metoki H, Asayama K, Obara T, Hashimoto J, Totsune K, Hoshi H, Satoh H, Imai Y (2005). Prognosis of “masked” hypertension and “white-coat” hypertension detected by 24-h ambulatory blood pressure monitoring 10-year follow-up from the Ohasama study. J Am Coll Cardiol.

[28] Chobanian AV, Bakris GL, Black HR, Cushman WC, Green LA, Izzo JL, Jones DW, Materson BJ, Oparil S, Wright JT, Roccella EJ (2003). The Seventh Report of the Joint National Committee on Prevention, Detection, Evaluation, and Treatment of High Blood Pressure: the JNC 7 report. JAMA.

[29] Clement DL, De Buyzere ML, De Bacquer DA, de Leeuw PW, Duprez DA, Fagard RH, Gheeraert PJ, Missault LH, Braun JJ, Six RO, van Der Niepen P, O'Brien E (2003). Prognostic value of ambulatory blood-pressure recordings in patients with treated hypertension. N Engl J Med.

[30] Labonte ME, Cyr A, Baril-Gravel L, Royer MM, Lamarche B (2012). Validity and reproducibility of a web-based, self-administered food frequency questionnaire. Eur J Clin Nutr.

[31] Bouchard C, Tremblay A, Leblanc C, Lortie G, Savard R, Theriault G (1983). A method to assess energy expenditure in children and adults. Am J Clin Nutr.

[32] Kuvin JT, Patel AR, Sliney KA, Pandian NG, Sheffy J, Schnall RP, Karas RH, Udelson JE (2003). Assessment of peripheral vascular endothelial function with finger arterial pulse wave amplitude. Am Heart J.

[33] Moerland M, Kales AJ, Schrier L, van Dongen MG, Bradnock D, Burggraaf J (2012). Evaluation of the EndoPAT as a Tool to Assess Endothelial Function. Int J Vasc Med.

[34] German JB, Gibson RA, Krauss RM, Nestel P, Lamarche B, van Staveren WA, Steijns JM, de Groot LC, Lock AL, Destaillats F (2009). A reappraisal of the impact of dairy foods and milk fat on cardiovascular disease risk. Eur J Nutr.

